# Multidrug-Resistant *Campylobacter coli* in Men Who Have Sex with Men, Quebec, Canada, 2015

**DOI:** 10.3201/eid2209.151695

**Published:** 2016-09

**Authors:** Christiane Gaudreau, Pierre A. Pilon, Jean-Loup Sylvestre, France Boucher, Sadjia Bekal

**Affiliations:** Université de Montréal, Montreal, Quebec, Canada (C. Gaudreau, P.A. Pilon, S. Bekal);; Centre Hospitalier de l’Université de Montréal, Montreal (C. Gaudreau, F. Boucher);; Centre Intégré Universitaire de Santé et de Services Sociaux du Centre-Sud-de-l’île-de-Montréal, Montreal (P.A. Pilon, J.-L. Sylvestre);; Laboratoire de Santé Publique du Québec/Institut National de Santé Publique du Québec, Sainte-Anne-de-Bellevue, Québec, Canada (S. Bekal)

**Keywords:** Campylobacter coli, emergence, antimicrobial resistance, drug resistance, men, Québec, homosexuality, men who have sex with men, Canada, bacteria, enteric infections, STIs

**To the Editor:** In 2015, an outbreak of multidrug-resistant *Campylobacter coli* was documented in Montreal, Quebec, Canada. We report results of an epidemiologic and molecular investigation suggesting a sexually transmitted enteric infection among men who have sex with men (MSM).

The ethics committee of Centre Hospitalier de l’Université de Montréal approved the research. During January 14–February 7, 2015, six men 35–62 years of age were documented with an enteric, erythromycin-, tetracycline- and ciprofloxacin-resistant *C. coli* pulsovar 15 infection. All 6 men had diarrhea; 5 had abdominal pain; 1 had fever >39°C; 1 had blood in feces; and 1 had vomiting. No extraintestinal focus was documented in these patients. 

Five men were evaluated in the outpatient clinic or emergency department; 1 man was hospitalized for 3 days. Five patients were treated with an antimicrobial agent. Three were treated orally for 4–7 days: 1 with ciprofloxacin, 1 with azithromycin, and 1 with both drugs. One patient was treated for 3 days with intravenous ceftriaxone and vancomycin followed by 10 days of amoxicillin for simultaneous *Streptococcus pneumoniae* septicemia. One man was treated with 1 intramuscular ceftriaxone dose, doxycycline for 21 days, and intravenous ertapenem for 3 days for proctitis and enterocolitis. All patients recovered with treatment (in vitro susceptible or resistant agent) or without treatment.

The 6 men reported to be MSM. The week before symptom onset, 4 men reported having had unprotected sex, 2 in bathhouses. Before the *C. coli* incubation period and after the outbreak started, 1 of these 2 men had traveled to the Caribbean but did not have sexual relations there. These men were not explicitly linked to each other. Five men were HIV positive; 1 was HIV-negative. The 5 HIV-positive men had CD4 counts ranging from 210 to 1,150 × 10^6^ cells/L and HIV viral load of <40 copies/mL. Since 2010, the 6 men had 15 documented sexually transmitted infections (STIs) other than HIV, 1–3 (median 3) STIs per patient: 4 *Treponema pallidum* infections; 3 *Chlamydia trachomatis* infections (1 rectal *C. trachomatis* serovar L2b, a lymphogranuloma venereum agent); 4 *Neisseria gonorrhoeae* infections; 3 *Shigella* spp. infections; and 1 *C. jejuni* infection.

The Laboratoire de Santé Publique du Québec (LSPQ, Sainte-Anne-de-Bellevue, QC, Canada) confirmed the 6 *C. coli* infections using *cpn60* gene sequencing (*1*). Drug susceptibility testing was done by using disk diffusion method for nalidixic acid and Etest (AB Biodisk, Solna, Sweden) for 12 other agents (*1*–*3*).The susceptibility and resistance breakpoints were Clinical and Laboratory Standards Institute *Campylobacter*, *Enterobacteriaceae*, and other breakpoints as reported (*1*–*4*). The 6 *C. coli* pulsovar 15 were resistant to erythromycin, azithromycin, clarithromycin, clindamycin, tetracycline, ciprofloxacin, nalidixic acid, ampicillin, and cefotaxime. All isolates were susceptible to amoxicillin/clavulanic acid, imipenem, ertapenem, and gentamicin. The 6 isolates were β-lactamase positive in <1 min with nitrocefin disk. Pulsed-field gel electrophoresis, done at LSPQ as described by PulseNet Canada procedures (*1*), showed that the 6 isolates presented the same pattern with both *Sma*I and *Kpn*I enzymes designed pulsovar 15 (Figure).

**Figure:**
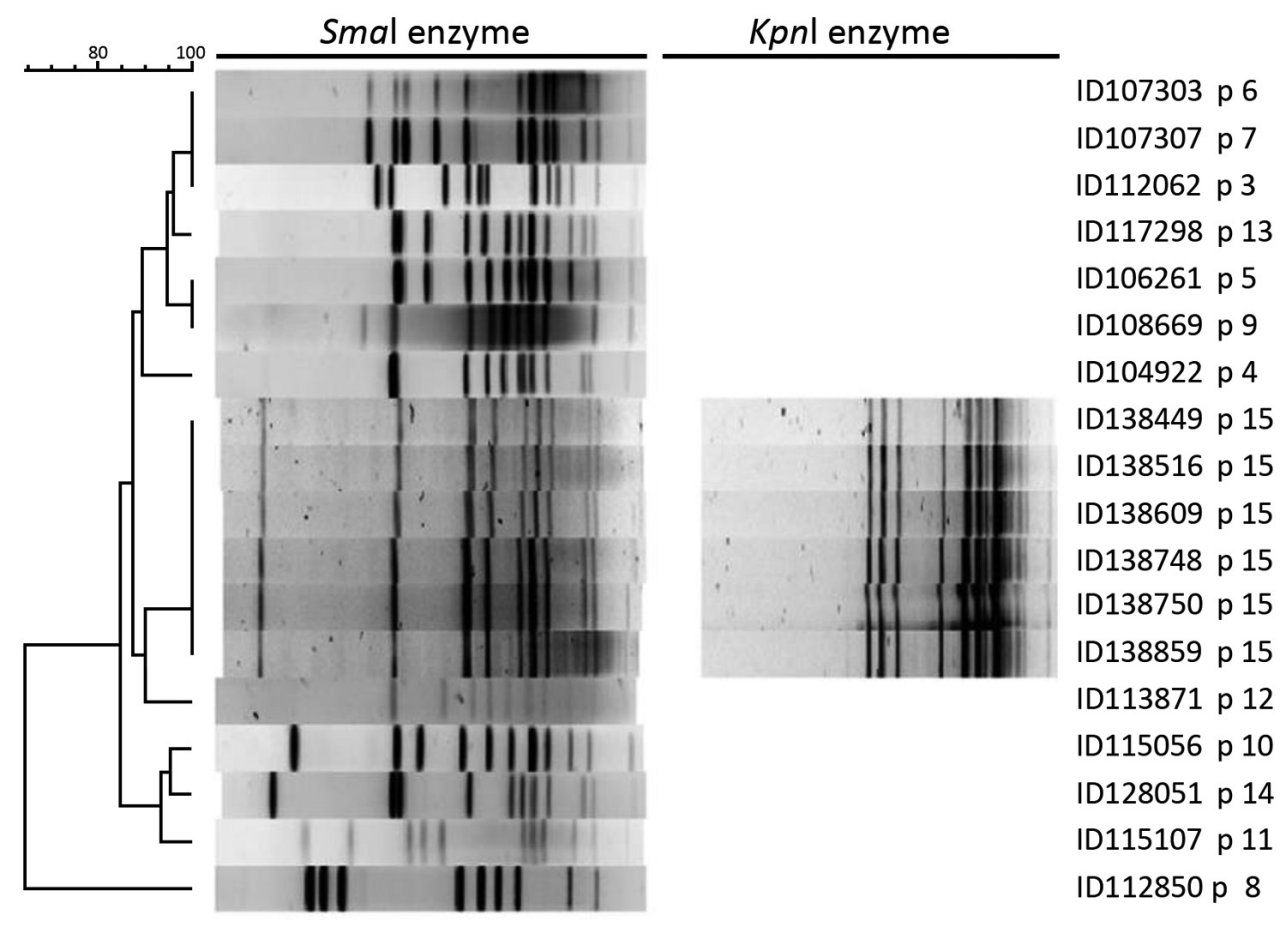
Pulsed-field gel electrophoresis patterns of *Campylobacter coli* with *Sma*I (18 isolates) and *Kpn*I (6 isolates) enzymes tested in study of *C. coli* outbreak among 6 men in Quebec, Canada, 2011–2015. p, pulsovar. Scale bar indicates percentage similarity.

These phenotypic, epidemiologic, and molecular data confirmed a cluster of an erythromycin-, tetracycline-, and ciprofloxacin-resistant *C. coli* pulsovar 15 infections in Montreal, Quebec, Canada, during January–February 2015. Epidemiologic data suggested enteric STIs. All 6 patients reported being MSM, 4 reported having unprotected sex the week before symptom onset; 5 were HIV-positive; the 6 men had 15 other STIs; and no food was suspected to be the source of the infection. 

*Campylobacter* is an important human enteropathogen bacterium, and *C. coli* is the second most frequently reported species (*4*–*6*). Few *C. coli* clusters have been reported, and the outbreaks caused by this *Campylobacter* species might be underestimated (*1*,*7*). At the LSPQ, a high heterogeneity was documented in *C. coli* isolates characterized routinely from suspected outbreaks during 2011–2015 (Figure) (*1*; this study). The erythromycin, tetracycline, and ciprofloxacin susceptibilities were epidemiologic markers in this study and in previous studies (*1*,*8*). The presence of a strong β-lactamase with resistance to ampicillin was also a marker in this study; epidemic *C. jejuni* and *C. coli* isolates were β-lactamase negative with susceptibility to ampicillin in previous outbreaks in MSM (*1*,*8*). Higher proportions of *C. coli* isolates are erythromycin- and multidrug-resistant than are *C. jejuni* isolates (*4*,*6*). When indicated, the proper antimicrobial treatment of enteric erythromycin- and ciprofloxacin-resistant *Campylobacter* spp. is not known because no clinical studies have been done for infections with such isolates, but tetracycline or amoxicillin/clavulanic acid can be used if isolates are susceptible in vitro (*1*,*8*; this study).

MSM should be counseled about preventing STIs, including enteric infections. Barriers should be used during genital, oral, and anal sex, and genital and hand washing before and after sex should be done (*9*,*10*). Our study increases evidence of clusters of *Campylobacter* STIs in MSM (*1*,*8*).
